# Hypermethylation of the *VTRNA1-3* Promoter is Associated with Poor Outcome in Lower Risk Myelodysplastic Syndrome Patients

**DOI:** 10.3390/genes6040977

**Published:** 2015-10-14

**Authors:** Alexandra Søgaard Helbo, Marianne Treppendahl, Derya Aslan, Konstantinos Dimopoulos, Cecilie Nandrup-Bus, Mette Skov Holm, Mette Klarskov Andersen, Gangning Liang, Lasse Sommer Kristensen, Kirsten Grønbæk

**Affiliations:** 1 Department of Hematology, Rigshospitalet, University Hospital Copenhagen, Blegdamsvej 9, 2100 Copenhagen Ø, Denmark; E-Mails: alexandra.soegaard@regionh.dk (A.S.H.); marianne.treppendahl@gmail.com (M.T.); qcl997@alumni.ku.dk (D.A.); konstantinos.dimopoulos@regionh.dk (K.D.); cecilie.nandrup-bus@regionh.dk (C.N.-B.); lasse.sommer.kristensen@regionh.dk (L.S.K.); 2 Department of Hematology, Aarhus University Hospital, Tage Hansens Gade 2, 8000 Aarhus C, Denmark; E-Mail: mettehlm@rm.dk; 3 Department of Clinical Genetics, Rigshospitalet, University Hospital Copenhagen, Blegdamsvej 9, 2100 Copenhagen Ø, Denmark; E-Mail: mette.klarskov.andersen@regionh.dk; 4 Department of Biochemistry and Molecular Biology, Norris Comprehensive Cancer Center, Keck School of Medicine, University of Southern California, 1441 Eastlake Avenue, Los Angeles, CA 90089, USA; E-Mail: gliang@usc.edu; 5 Danstem, University of Copenhagen, Blegdamsvej, 2200 Copenhagen N, Denmark

**Keywords:** DNA methylation, non-coding RNA, Myelodysplastic syndrome, Azanucleosides, vault RNA

## Abstract

Myelodysplastic syndrome (MDS) is a heterogeneous group of clonal hematopoietic disorders. MDS is frequently associated with deletions on chromosome 5q as well as aberrant DNA methylation patterns including hypermethylation of key tumor suppressors. We have previously shown that hypermethylation and silencing of the non-coding RNA *VTRNA2-1* are correlated with poor outcomes in acute myeloid leukemia patients. In this study, we find that *VTRNA1-2* and *VTRNA1-3*, both located on chromosome 5q, can be regulated and silenced by promoter DNA methylation, and that the hypomethylating agent 5-aza-2-deoxycytidine causes reactivation these genes. In normal hematopoiesis, we find that vault RNAs (vtRNAs) show differential methylation between various hematopoietic cell populations, indicating that allele-specific methylation events may occur during hematopoiesis. In addition, we show that *VTRNA1-3* promoter hypermethylation is frequent in lower risk MDS patients and is associated with a decreased overall survival.

## 1. Introduction

Low risk myelodysplastic syndrome (MDS) is a heterogeneous group of clonal diseases characterized by ineffective hematopoiesis due to increased apoptosis and differentiation block of early progenitors, resulting in cytopenia in the myeloid lineages [1]. It has recently been proven that lower risk MDS is derived from the hematopoietic stem cell (HSC) compartment and that the disease is propagated in successive progenitor cells [2,3]. The most commonly used prognostic measure in MDS is the International Prognostic Scoring System (IPSS) [4], which accounts for the percentage of bone marrow blasts, number of peripheral blood cytopenias and karyotype. The IPSS allows differentiation of lower risk (low and intermediate-1 (INT-1) IPSS scores) from higher risk (intermediate-2 (INT-2) and high risk IPSS scores) MDS patients in terms of survival and risk of leukemic transformation.

Promoter DNA methylation silences gene expression, and altered DNA methylation patterns are involved in both the initiation and propagation of cancer [5,6]. MDS blasts have high rates of mutations in epigenetic modifiers and exhibit altered DNA methylation patterns [7,8,9,10]. Azanucleosides (aza) are inhibitors of DNA methylation and may thereby induce gene expression. Two aza analogs are FDA approved for the treatment of MDS (5-azacytidine and 5-aza-2-deoxycytidine (decitabine; 5-Aza-CdR)) [11] and are effective in improving outcomes for MDS patients [12,13,14,15]. However, there is a strong need for prognostic and predictive biomarkers as responses take several months to occur and not all patients respond [16].

A tumor suppressor has long been sought in the chromosomal region 5q31-33, which is commonly deleted in MDS [17,18], and although investigations in recent years have identified several haploinsufficient candidate genes, with rare biallelic disruptions [19], there is still discussion of which genes are the most biologically relevant targets of these deletions in MDS. Previous studies have implicated disruption of non-coding RNAs (ncRNAs) in this region in the pathogenesis of MDS and other bone marrow failure diseases [20,21]. We have previously reported that the vault RNA (vtRNA) *VTRNA2-1* (also called nc886) is regulated by DNA methylation and is involved in the clinical outcome of acute myeloid leukemia (AML) patients [22]. *VTRNA2-1* has also been suggested to be a tumor suppressor in other cancers [23,24,25,26,27]. Interestingly, the other vtRNAs (*VTRNA1-1*, *VTRNA1-2* and *VTRNA1-3*) are located in close proximity to *VTRNA2-1* on chromosome 5q31.3 (previously reported on 5q33.1 [28]). The cytosolic fraction of vtRNAs has been suggested to be involved in miscellaneous functions including the innate immune response, exosomes, apoptosis, to function as microRNAs and has been linked to chemotherapy resistance [29,30,31,32,33,34]. Given its localization in a commonly deleted region, and the putative tumor suppressor properties associated with *VTRNA2-1*, we suggested that *VTRNA1-1*, *1-2* and *1-3* may also be potential tumor suppressors and investigated whether DNA methylation could co-regulate their expression and be involved in the pathogenesis of MDS.

Here, we show that *VTRNA1-2* and *VTRNA1-3* can be regulated by promoter DNA methylation, and that silencing can be reversed by 5-aza-CdR treatment. In addition, we find that *VTRNA1-3* is silenced by DNA methylation in a human leukemia cell line, but unmethylated in CD34+ HSCs from healthy controls, indicating cancer-specific silencing. Lastly, we find that *VTRNA1-3* promoter methylation is associated with poor outcomes in lower risk MDS patients, indicating that this ncRNA may be a potential tumor suppressor in this patient subgroup.

## 2. Materials and Methods

### 2.1. Patients and Healthy Donors

Bone marrow mononuclear cells (BM-MNCs) or unseparated bone marrow cells were obtained from 140 MDS patients at the time of diagnosis. The patient samples were collected at the Department of Hematology, Rigshospitalet, Copenhagen, and Aarhus University Hospital, Aarhus, between 2008 and 2013. Patients were diagnosed according to the World Health Organization (WHO) criteria [35], and the International Prognostic Scoring System (IPSS) [4] was used to stratify the MDS patients into risk-groups.

In addition, peripheral blood MNCs (PBMCs) were collected from 20 healthy donors (with no hematological or other known disease) after informed consent. We additionally collected BM-MNCs from seven of these donors. The ethical committees of all participating institutions approved the study. The study was approved by the ethical committee for the Capital Region of Denmark (H-D-2009-003) and the Danish Data Protection Agency (30-1419) and conducted in accordance with the tenants of Helsinki.

### 2.2. Cell Culture and Drug Treatments

HL60 cells were cultured in a RPMI 1640 medium with Glutamax-1, all supplemented with 10% fetal bovine serum (FBS), 100 U/mL penicillin and 100 µg/mL streptomycin. As previously described, HL60 cells were treated with 5-aza-CdR and harvested on day 2 and 8 after treatment [36]. In short, cells were seeded and received 5-aza-CdR the following day. Twenty-four hours after 5-aza-CdR, the drug was removed from the media and cells were cultured according to regular conventions until harvested.

### 2.3. DNA Extraction and Bisulfite Conversion

DNA was extracted using a Gentra Puregene Cell Kit (Qiagen, Valencia, CA, USA) or the AllPrep DNA/RNA mini kit (Qiagen) according to manufacturer’s instructions. DNA was bisulfite converted using the EZ DNA Methylation Kit (Zymo, Irvine, CA, USA) according to the manufacturer’s instructions.

### 2.4. RNA Extraction and Reverse Transcriptase Quantitative PCR (RT-qPCR)

RNA from cell lines was isolated using Trizol and reverse transcribed using SuperScript III reverse transcriptase (Invitrogen, Waltham, MA, USA) with both Oligo-dT (Invitrogen) and random hexamers (Promega, Madison, WI, USA) for all samples. RT-qPCR was performed using custom primers and TaqMan probes (Applied Biosystems, Grand Island, NY, USA) for vtRNA transcripts, whereas GAPDH was quantified using SYBR green (Roche, Basel, Switzerland). Primer and probe sequences are listed in Table S1.

### 2.5. Chromatin Immunoprecipitation (ChIP)

ChIP was performed as previously described [37]. Antibodies against IgG (2729S) were purchased from Cell Signaling and H3K4me3 (cat. No. 39160) from Active Motif. Quantification of immunoprecipitated DNA was performed by RT-qPCR using SYBR green (Roche). Primer sequences are listed in Table S1.

### 2.6. DNA Methylation Analyses

#### 2.6.1. Bisulfite-Sequencing

To analyze the methylation status of individual DNA molecules, bisulfite-treated genomic DNA was PCR amplified and cloned into the pCR2.1 vector using the TOPO-TA cloning kit (Invitrogen). Colonies were screened for the respective inserts. Plasmid DNA was amplified using Templiphi (GE Healthcare, Bucks, UK). Plasmid DNA from individual clones was automatically sequenced using M13 primers by the commercial services of Eurofins. Primer sequences are listed in Table S1.

#### 2.6.2. Methylation-Sensitive Single-Nucleotide Primer Extension (MS-SNuPE)

Ms-SNuPE was performed as previously described [38]. Primer sequences are listed in Table S1.

#### 2.6.3. Pyrosequencing

The promoters of *VTRNA1* family genes are highly similar, and in order to ensure specificity for the *VTRNA1-3* promoter, we first performed an outer PCR, followed by a nested PCR to create a shorter fragment suitable for pyrosequencing. The outer PCR was performed using the GoTaq reagents (Promega), followed by the nested PCR using the PyroMark PCR kit (Qiagen) according to the manufacturer’s instructions. The region analyzed covers four CpG sites immediately upstream of the *VTRNA1-3* transcription start site (TSS), and the mean methylation value of these sites was calculated for each sample. Methylation analysis by pyrosequencing of the vtRNA1-3 promoter was carried out on a PyroMark Q24 (Qiagen) using the PyroMark Gold Q24 reagents (Qiagen) according to the manufacturer’s instructions. Primer sequences are listed in Table S1.

### 2.7. Survival Statistics

Association of *VTRNA1-3* methylation with other factors was evaluated using chi-square test or Fisher’s exact test. Five-year overall survival was calculated as the time from diagnosis to death or last follow-up. Survival curves were created with the Kaplan-Meier method and compared using the log-rank test. To evaluate the simultaneous effect of different factors in overall survival, we performed a multiple Cox-regression analysis with backward stepwise factor elimination. The level of significance in all tests was set as *p* < 0.05 and all *p*-values in this study are two-sided.

## 3. Results

### 3.1. VTRNAs Are Regulated by DNA Methylation

**Figure 1:**
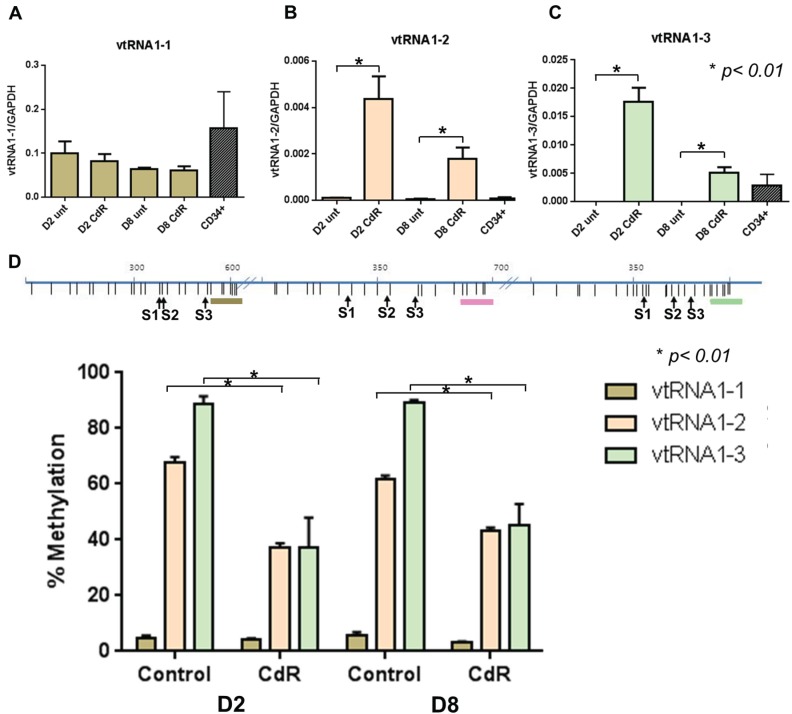
5-Aza-CdR causes demethylation and reactivation of *VTRNA1-2* and *VTRNA1-3*. HL60 cells were treated for 24 h with 5-aza-CdR and harvested on day 2 (D2) or day 8 (D8) after treatment. (**A**) Expression of *VTRNA1-1* in HL60 and normal CD34+ HSCs; (**B**) Expression of *VTRNA1-2* in HL60 and normal CD34+ HSCs; (**C**) Expression of *VTRNA1-3* in HL60 and normal CD34+ HSCs; (**D**) Methylation changes determined by Ms-SNuPE in HL60 cells during 5-aza-CdR treatment at *VTRNA1-1, 1-2* and *1-3* promoters. The top panel indicates location of CpG sites relative to vtRNA TSSs (brown *VTRNA1-1*, pink *VTRNA1-2* and mint *VTRNA1-3*) and S1, S2, and S3 indicate the specific CpG sites analyzed by Ms-SNuPE. Mean + SD shown for expression, mean + SEM for methylation data, *p*-values were calculated by Mann-Whitney U test.

We first treated the myeloid cancer cell line HL60 with 5-aza-CdR and examined the expression of the three vtRNA transcripts compared to the expression in normal CD34+ HSCs (Figure 1A–C). We observed that the expression of *VTRNA1-1* was unaltered by 5-aza-CdR treatment, while *VTRNA1-2* and *VTRNA1-3* were significantly upregulated following 5-aza-CdR treatment (Figure 1B,C).

Upregulation was highest at day 2, which may be explained by proliferation of the cells, where the drug was increasingly washed out of the cell population with time. We observed low expression of *VTRNA1-2* in normal CD34+ HSCs compared to *VTRNA1-3*, where the expression of *VTRNA1-3* in HL60 following 5-aza-CdR treatment exceeds or reaches the endogenous expression levels of *VTRNA1-3* in CD34+ cells (Figure 1B,C). We next analyzed the methylation status of vtRNA promoters in untreated *vs.* 5-aza-CdR treated HL60 cells (Figure 1D). As expected, we found that the *VTRNA1-1* promoter is unmethylated, while both *VTRNA1-2* and *VTRNA1-3* promoters are highly methylated in untreated HL60 cells (~70%–90% methylation) and demethylate (to ~40% methylation) subsequent to 5-aza-CdR treatment, corresponding to the upregulation in expression observed in Figure 1B,C. We furthermore observe that re-activation of *VTRNA1-2* and *VTRNA1-3* expression correlates to the deposition of the histone mark H3K4me3, which is a hallmark of active promoters (Figure S1), thereby further highlighting that 5-aza-CdR causes the observed reactivation of vtRNAs silenced by DNA methylation. Accordingly, this shows that *VTRNA1-2* and *VTRNA1-3* can be co-regulated by DNA methylation, and that high methylation levels results in the silencing of these genes.

### 3.2. VTRNAs Are Differentially Methylated in Healthy Donor Cell Populations

**Figure 2:**
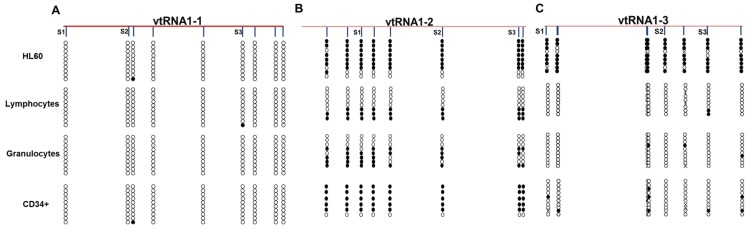
Cell type specific methylation patterns at vtRNA promoters shows that *VTRNA1-3* may be cancer specifically silenced. Bisulfite sequencing of vtRNA promoters in HL60 cells, healthy donor lymphocytes, granulocytes and CD34+ HSCs. (**A**) *VTRNA1-1* promoter; (**B**) *VTRNA1-2* promoter; and (**C**) *VTRNA1-3* promoter. The location of the Ms-SNuPE primers used in the previous analyses are shown as S1, S2 and S3.

Our results show that among the three *VTRNA1s*, *VTRNA1-3* was the only gene silenced in cancer cells and also expressed in healthy donor CD34+ cells. We hypothesized that the *VTRNA1-3* promoter is unmethylated in healthy hematopoietic cells, and we therefore examined the methylation status of *VTRNA* promoters in various cell populations from a healthy donor (Figure 2). The *VTRNA1-1* promoter is unmethylated in all cell types (Figure 2A), while the *VTRNA1-2* promoter is highly methylated in healthy donor CD34+ HCSs, similar to HL60 cells (Figure 2B).

Moreover, *VTRNA1-2* showed methylation of roughly half of the DNA molecules in granulocytes and lymphocytes. Note that individual DNA molecules were either fully methylated or unmethylated and do not exhibit partial methylation, indicating that *VTRNA1-2* may be allele-specifically methylated, as we previously observed for *VTRNA2-1* [22]. In contrast, the *VTRNA1-3* promoter was unmethylated in all healthy control cell types, with only a few single sites being methylated (Figure 2C). Based on these data we hypothesized that *VTRNA1-3* may be a candidate tumor suppressor as it is silenced by DNA methylation in cancer HL60 AML cells, but unmethylated in healthy donor hematopoietic cells.

### 3.3. VTRNA1-3 Promoter Methylation Associates with Poor Survival in Lower Risk MDS Patients

**Figure 3:**
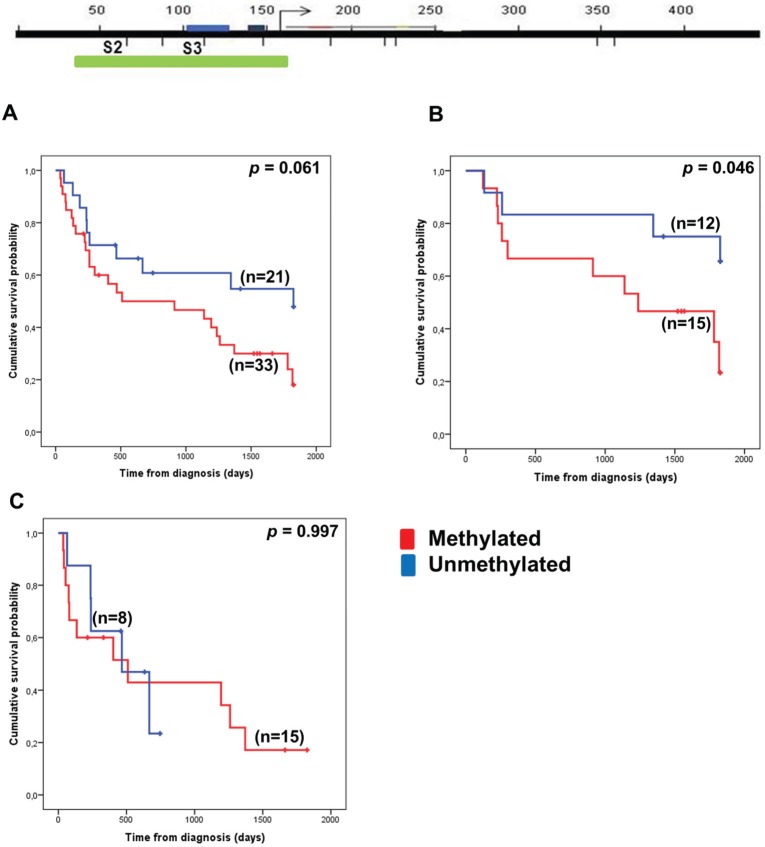
Methylation status of the *VTRNA1-3* promoter associates only with 5-year overall survival in the lower risk MDS patient subgroup. Hypermethylation of *VTRNA1-3* was defined as the occurrence of mean methylation levels ≥ 20% as determined by pyrosequencing. The top panel indicates the region analyzed by pyrosequencing (green box) relative to the TSS and the Ms-SNuPE primers used in the previous analyses (promoter elements are indicated by blue boxes). 5-Year overall survival according to *VTRNA1-3* methylation in (**A**) all MDS patients; (**B**) lower risk (LR and INT-1) patients; and (**C**) higher risk (INT-2 and HR) patients. A break in the line indicates a censored event.

As the region on 5q is of particular interest in MDS, we examined the role of *VTRNA1-3* promoter methylation in BM-MNCs from the time of diagnosis to survival outcome in a pilot cohort of MDS patients representing mixed IPSS groups (Figure 3).

**Figure 4:**
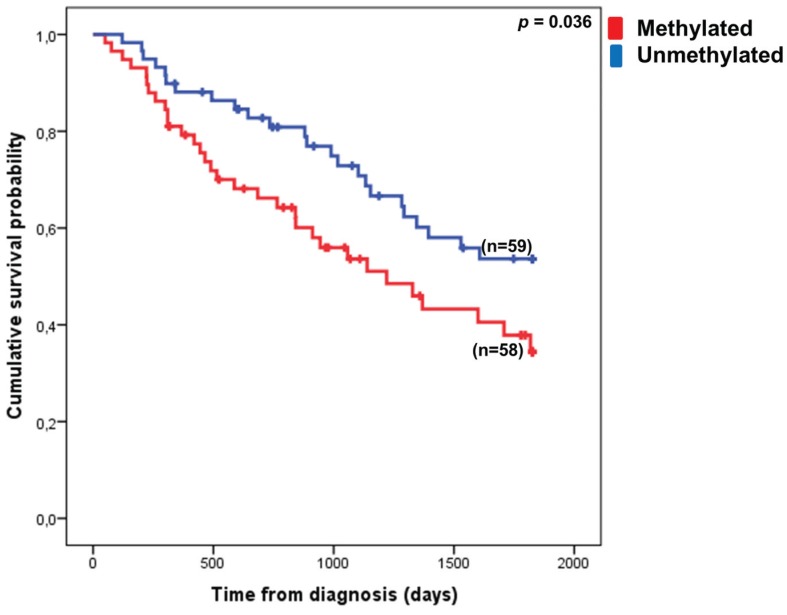
Hypermethylation of the *VTRNA1-3* promoter associates with poor outcome in lower risk MDS patients. Patients with <20% mean methylation of the *VTRNA1-3* promoter in their BM cells have a significantly better 5-year overall survival than patients exhibiting ≥20% mean methylation. A break in the line indicates a censored event.

We defined a hypermethylation cutoff as a mean methylation level of ≥20%. This was based on the mean *VTRNA1-3* promoter methylation of 20 healthy individuals plus two times the standard deviation (Figure S2). In the initial cohort of 54 MDS patients, our Kaplan-Meier analysis did not show a significant effect of *VTRNA1-3* promoter methylation on 5-year overall survival (Figure 3A). However, as MDS is a heterogeneous disease, we subdivided the cohort into lower risk (LR and INT-1 IPSS groups) and higher risk (INT-2 and HR IPSS groups) (Figure 3B,C), and performed the same analyses. Here, we observed a significant correlation between *VTRNA1-3* promoter hypermethylation and outcomes only in the lower risk patient subgroup (*p* *=* 0.046, Figure 3B). Lower risk MDS patients with a methylated *VTRNA1-3* promoter in BM cells had poorer survival rates than patients with an unmethylated *VTRNA1-3* promoter. This indicates that *VTRNA1-3* promoter hypermethylation might predict poor outcomes in lower risk MDS patients, and in order to validate this hypothesis, we increased the number of patients in the lower risk MDS cohort (Figure 4). The patient characteristics of the lower risk cohort are summarized in Table 1.

**Table 1:** Clinical characteristics of lower risk patients.

		n	%
Age	<65	20	17.1
	≥65	97	82.9
Sex	Male	59	54.1
	Female	50	45.9
IPSS score	0	61	60.3
	0.5–1	50	39.7

In our extended cohort, we found that *VTRNA1-3* hypermethylation is associated with poor outcome in lower risk MDS patients (*p* = 0.036). Log-rank analysis of other individual factors related to MDS revealed that age, blast count and cytogenetics were, as expected, also significant covariates in the 5-year overall survival of lower risk MDS patients (Table 2). Chi-square analysis revealed that there was no association between *VTRNA1-3* hypermethylation and any of the other covariates tested (age, sex, IPSS score, blast count groups and cytogenetic groups).

**Table 2:** Log-rank analysis for individual variables (covariates) and multiple Cox-regression analysis of all the included variables.

Covariate	Log-Rank Analysis				Multiple Cox Regression Analysis			
	Coefficient	HR	95% CI	*p*-value	Coefficient	HR	95% CI	*p*-value
Age (≥65 years)	1.286	3.617	1.305–10.030	0.008	1.506	4.508	1.373–14.801	0.013
Sex	−0.002	0.998	0.578–1.723	0.995	−0.268	0.765	0.416–1.405	0.388
*VTRNA1-3* methylation	0.562	1.755	1.031–2.985	0.037	0.360	1.434	0.801–2.566	0.225
IPSS score (0 *vs.* 0.5–1)	0.369	1.446	0.843–2.479	0.178	0.022	1.022	0.510–2.046	0.951
Blast count (<5% *vs.* 5%–10%)	1.098	2.999	1.397–6.437	0.003	1.310	3.705	1.574–8.722	0.003
Cytogenetics (good *vs.* intermediate/poor)	0.854	2.350	1.236–4.465	0.007	1.195	3.303	1.662–6.564	0.001

However, when the relative impact of *VTRNA1-3* promoter methylation on 5-year overall survival was evaluated by multiple Cox-regression analysis, *VTRNA1-3* promoter methylation was not found to be an independent prognostic factor (*p* = 0.225, Table 2), while age, blast count and cytogenetic scores retained their independent prognostic value. Thus, our data indicate that hypermethylation of *VTRNA1-3* is associated with poor outcomes of lower risk MDS patients, but is not an independent prognostic factor.

## 4. Discussion

MDS is a heterogeneous disease and it appears to be increasingly likely that variable factors are involved in the pathogenesis of either lower or higher risk MDS, respectively. This may also explain why numerous candidate tumor suppressors in the 5q31-33 region have been reported, but few have been accepted as universally valid prognostic factors in MDS. In addition, it is evident that non-coding RNAs are deregulated in carcinogenesis, often through epigenetic mechanisms [39,40]. Our study highlights the above observations, as we show that vtRNAs are transcriptionally regulated by DNA methylation and that hypermethylation of the *VTRNA1-3* promoter is associated with poor outcomes specifically in lower risk MDS patients.

While all DNA molecules are fully methylated at the *VTRNA1-2* promoter in healthy donor CD34+ HSCs, we observe a decrease to 50% methylated molecules in the differentiated lymphocyte and granulocyte cell populations. We previously reported that *VTRNA2-1* is mono-allelically methylated in 75% of the Danish population [22], but this was consistent in all blood cell populations analyzed. Methylation of *VTRNA2-1* was later suggested to be linked to the maternal allele [26]. It would therefore be of interest to investigate if allele-specific methylation of the *VTRNA1-2* locus occurs during, or as a consequence of HSC differentiation, and if this is linked to the maternal or paternal allele.

The exact functions of vtRNAs have been a matter of much debate. While *VTRNA1-3* promoter methylation it is not an independent prognostic factor, we propose that *VTRNA1-3* may be involved in maintenance of normal HSC function, and that its silencing by DNA methylation may be involved in the pathogenesis of lower risk MDS. Lower risk MDS is indeed characterized by excessive bone marrow apoptosis [41], and, interestingly, it was recently shown that *VTRNA1-1* is involved in resistance to apoptosis during infection [29,31]. Thus, it is likely that the downregulation of *VTRNA1s* by deletion, methylation or other molecular mechanisms may contribute to the increased apoptosis observed in the bone marrow of these patients.

Interestingly, it has previously been suggested that *VTRNA2-1*’s putative tumor suppressor function is linked to phosphorylation of the RNA sensitive protein kinase receptor (PKR) [22,42,43], a key event in interferon signaling. It was suggested that *VTRNA2-1* could modulate PKRs sensitivity to double stranded RNAs (dsRNAs), and loss of *VTRNA2-1* was critical for tumorigenesis. It is currently unknown if the *VTRNA1s* hold similar properties. However, two recent studies showed that low dose aza treatment of cancer cells results in the demethylation and upregulation of endogenous retroviruses (ERVs), which form dsRNA that via the viral defense pathway triggers an interferon response and hence apoptosis through an immune mediated mechanism [44,45]. Since we showed that 5-aza-CdR can re-activate *VTRNA1-3* through demethylation of the promoter, it could of interest to investigate the effect of 5-aza-CdR on *VTRNA1-3* promoter methylation and interferon signaling *in vivo* in samples obtained from patients during treatment. Furthermore, as only around 50% of MDS patients respond to aza treatment, a predictive biomarker for treatment response is warranted [16], and it may be of interest to investigate if *VTRNA1-3* methylation could be such a biomarker.

In summary, our data suggest that deregulated vault expression is involved in the pathogenesis of lower risk MDS. We find that *VTRNA1-3* methylation is associated with poor prognosis, and could potentially be associated with a tumor suppressor function in this subset of the disease, although functional experiments are warranted in order to validate such a cellular function. Future studies will aim at identifying the exact role of this vtRNA in normal and malignant hematopoiesis.

## 5. Conclusions

We provide the first study of vtRNA methylation in the pathogenesis of lower risk MDS, and found that *VTRNA1-2* and *VTRNA1-3* are regulated by DNA methylation in HL60 cells. Furthermore, we observed that their expression can be induced through 5-aza-CdR induced promoter demethylation. While not an independent prognostic factor, we find that hypermethylation of the *VTRNA1-3* promoter is associated with a decreased 5-year overall survival specifically in lower risk MDS.

## Supplementary Files

Supplementary File 1
