# Supplementary material for: Hypermethylation of the VTRNA1-3 Promoter is Associated with Poor Outcome in Lower Risk Myelodysplastic Syndrome Patients
Source: Genes (Basel). 2015 Oct 14;6(4):977–90. doi: 10.3390/genes6040977 (PMC4690025; doi:10.3390/genes6040977)
Supplement: Supplementary File 1 [file genes-06-00977-s001.pdf]

Supplementary Materials

Table S1. Primer sequences.

	Forward primer (5'–3')	Reverse primer (5'–3')	Probe (5'–3')
RT-qPCR primers			
vtRNA1-1	TGGCTTTAGCTCAGCGGTTAC	TCTCGAACAACCCAGACAGGTT	TCGACAGTTCTTTAATTGA
vtRNA1-2	TGGCTTTAGCTCAGCGGTTAC	GGTCTCGAACCACCCAGAGA	TCGAGTACATTGTAACCAC
vtRNA1-3	TGGCTTTAGCTCAGCGGTTAC	GGGTCTCGAACAACCCAGAGA	CGTGTCATCAAACCAC
GAPDH	aggtggtctcctctgacttcaacag	Gaaatgagcttgacaaagtggtcgt	NA
Bisulfite sequencing primers			
vtRNA1-1	AAGAGTTTTTAGGGATTAGGAA AGGT	CAACCCAAACAAATTACTTATTTCAATTAAAAAACTA	NA
vtRNA1-2	TTTGGTTTAAGTATTAGTTGTAAA AGT	TCCTTCTCAACAATCACCTA	NA
vtRNA1-3	AGTTGTGGATTAGGTGATTGTT	CTAAACTAAAACCAACCCAAAAATTA	NA
Ms-SNuPE primers			
vtRNA1-1	AAGAGTTTTTAGGGATTAGGAA AGGT	CAACCCAAACAAATTACTTATTTCAATTAAAAAACTA	AGGGATTAGGAAAGGTTA
			ATAATTTTTTAATTGTTTGGAGGT
			AGGTTTTTTTTATTAGTTAATAAAATATAATT
vtRNA1-2	TTTGGTTTAAGTATTAGTTGTAAA AGT	TCCTTCTCAACAATCACCTA	TGGTTGTTTTGGGAG
			GAAAATAAGTTAGGTATATAATTG
			GTATATTTTAATTGTTTGGAGG
vtRNA1-3	AGTTGTGGATTAGGTGATTGTT	CTAAACTAAAACCAACCCAAAAATTA	GAGGAGAGAATAAAT
			GATTGGATAGTTTAGGT
			TTGTTTTGATTTATTTTTTGTGA
Pyrosequencing primers			
vtRNA1-3 outer	TGTATGTTTTTAGATGGATATTTT	AACATTACTAAACACTAAAAAAACTT	NA
vtRNA1-3 nested	TGTATGTTTTTAGATGGATATTTT	AAAACCAACCCAAAAATTACTTT	NA

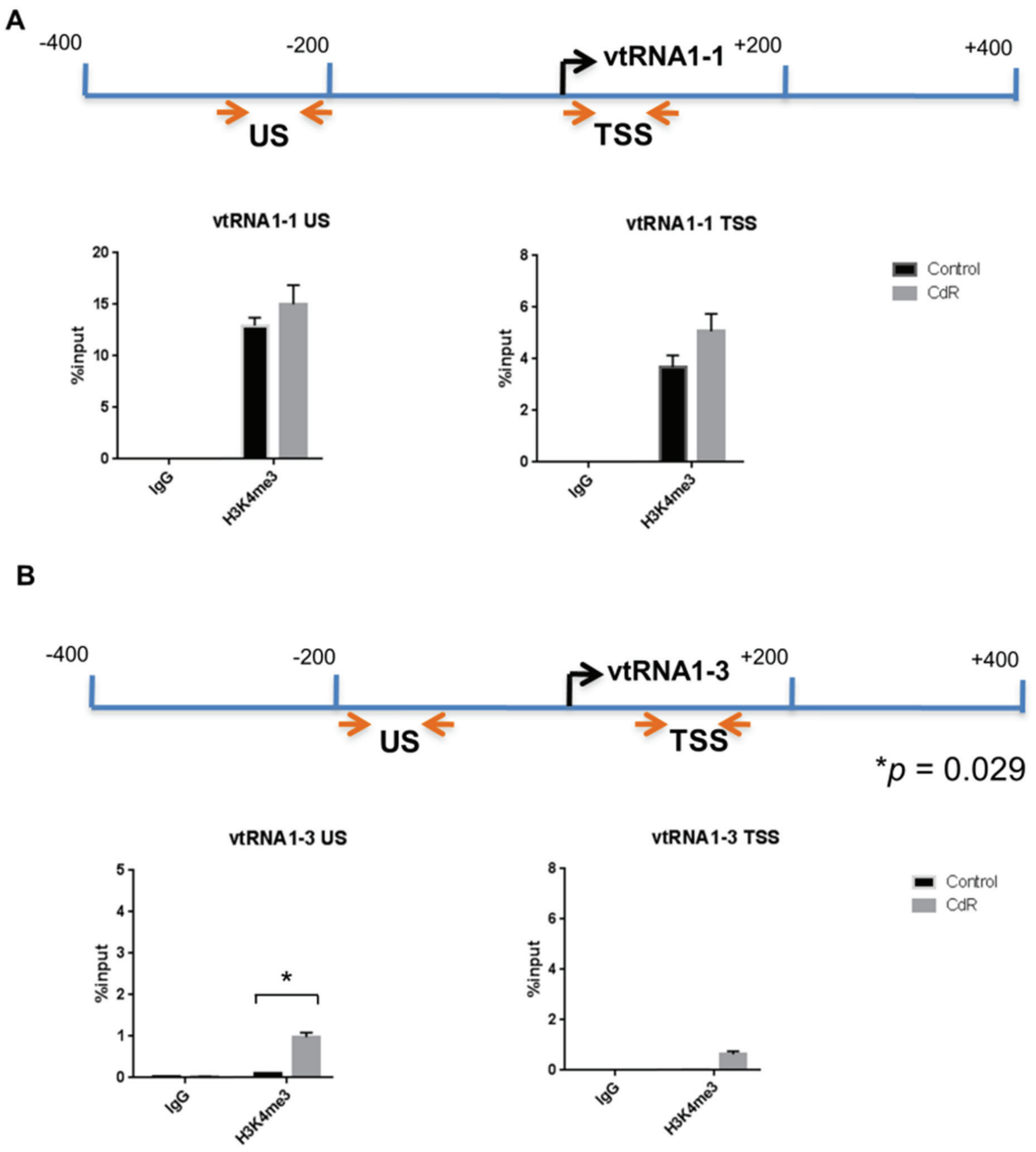

Figure S1. Re-activation of *VTRNA1-3* correlates with increased H3K4me3. HL60 cells were treated for 24 h with 5-Aza-CdR and harvested on D2 and ChIP against IgG and H3K4me3 was performed at (**A**) *VTRNA1-1* promoter (left panel) and TSS (right panel); and (**B**) *VTRNA1-3* promoter (left panel) and TSS (right panel). As *VTRNA1-1* is unmethylated, active in controls and unaffected by 5-Aza-CdR, we expect no change in H3K4me3. Significant differences are indicated by * (student's *t*-test). Mean + SD shown.

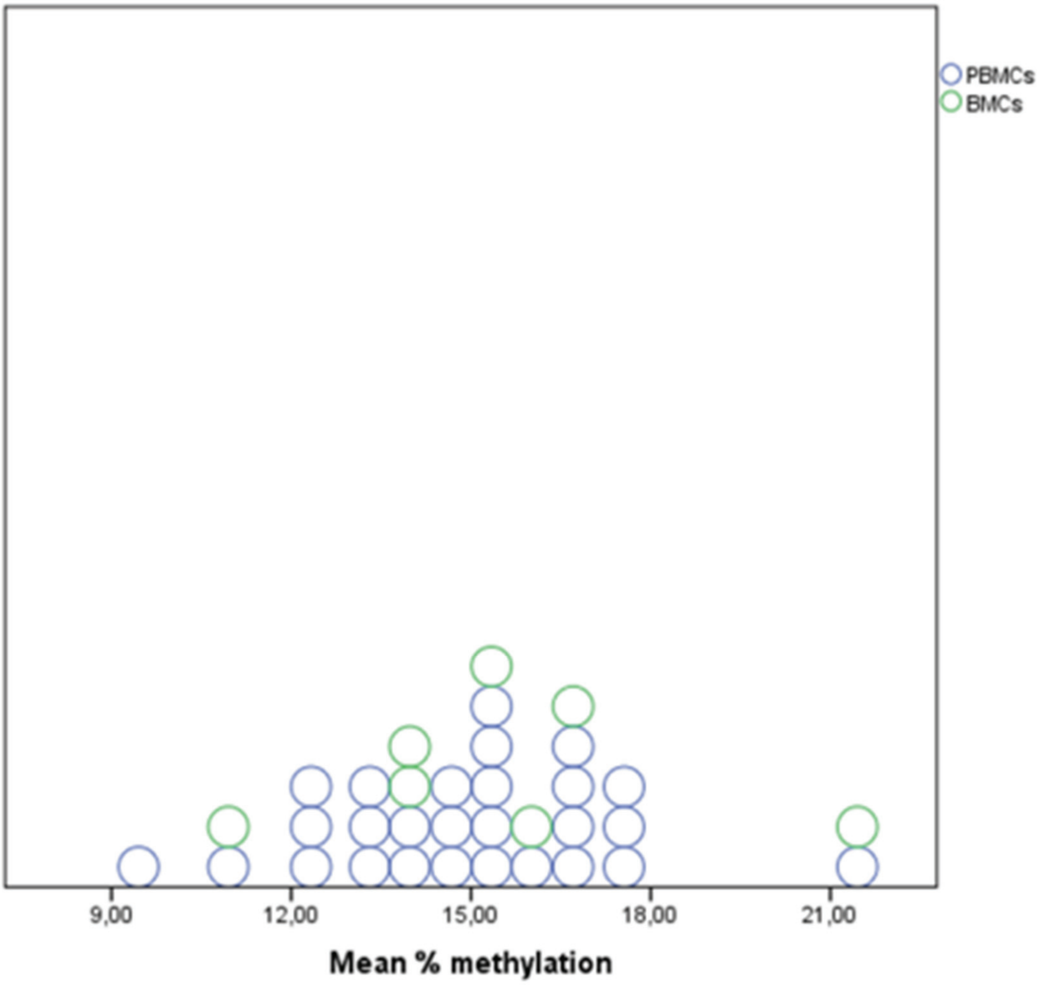

Figure S2. Methylation of the *VTRNA1-3* promoter in healthy donors. The mean methylation levels determined by pyrosequencing in individual donor's MNCs from blood or bone marrow are shown. We collected both blood and bone marrow MNCs from 7 of the 20 healthy donors used in this study. As the *VTRNA1-3* promoter methylation levels for blood and bone marrow MNCs are identical within the individual donors, the mean methylation was calculated from the blood MNCs methylation values. The cutoff was defined as mean *VTRNA1-3* methylation + 2*SD = ≥ 20%.

© 2015 by the authors; licensee MDPI, Basel, Switzerland. This article is an open access article distributed under the terms and conditions of the Creative Commons Attribution license (http://creativecommons.org/licenses/by/4.0/).
